# Severity of Neuropathy-Related Disability and Associated Factors of Diabetic Peripheral Neuropathy in a Tertiary Healthcare Center: A Comparative Cross-Sectional Study

**DOI:** 10.7759/cureus.55568

**Published:** 2024-03-05

**Authors:** Daris Francis, Kandaswami Kotteeswaran, Pramod Padinhare Veedu

**Affiliations:** 1 Department of Physiotherapy, Saveetha College of Physiotherapy, Saveetha Institute of Medical and Technical Sciences (SIMATS), Thandalam, IND; 2 Department of Physiotherapy, Lourde Institute of Allied Health Sciences, Taliparamba, IND

**Keywords:** vibration perception threshold, neuropathy disability score, diabetic nephropathy, diabetes mellitus, ankle dorsiflexion

## Abstract

Background and objectives: The widespread presence of diabetic peripheral neuropathy (DN) and its related variables among diagnosed diabetes mellitus (DM) patients in Kerala lacks sufficient evidence. The objective of this study was to evaluate the frequency of DN and its related risk variables among those with DM who were receiving care at a tertiary health institution in Kerala.

Materials and methods: This study was conducted in a tertiary health center in Kerala using a cross-sectional design. The diagnosis of diabetes was established by assessing the glycated hemoglobin A1c (HbA1c) level and the fasting blood glucose (FBG) level. A validated survey questionnaire was employed to gather demographic data as well as the medical history of DM and associated ailments. A thorough physical examination, BMI, and blood pressure were recorded. Dermatological, musculoskeletal, neurological, and vascular assessments were conducted. The subjects were assessed for neuropathy using a neuropathy disability score (NDS). Consequently, those who met the criteria for DM were classified into two groups: those with neuropathy and those without neuropathy. All research participants underwent laboratory examinations such as blood lipid profiles, HbA1c, and vitamin B12 complex concentrations.

Results: The study included 200 DM patients; 120 men and 80 women. Study participants were 40-70 years old, with a median age of 53. The prevalence of DN significantly increased with age (p<0.001). The longer a patient had DM, the higher the prevalence of DN, and this variance in percentage was statistically significant (p<0.009) with an OR (odds ratio) of 9.246. A statistically significant 81 (40.5%) of participants graduated, compared to 119 (59.5%) with only higher secondary education or less (OR = 2.042; p = 0.014). Approximately 107 (53.5%) of individuals earned an income under two lakhs, and this disparity was deemed statistically significant (p = 0.003) with an OR of 2.357. In the group of individuals being studied, 53 (26.5%) of them experienced a decrease in pressure sensation, 47 (23.5%) had a decrease or absence of vibration perception, 48 (24%) had an absence or decreased pinprick response, and 46 (23%) had an absent ankle reflex. The study found that the most commonly reported symptoms were tingling (n = 44; 22%), numbness (n = 42; 21%), burning (n = 37; 18.5%), pricking (n = 29; 14.5%), and pain (n = 27; 13.5%). A strong association was found between DN and glycemic status, namely, FBG levels exceeding 140 mg/dL (OR = 4.511, p < 0.001) and HbA1c levels exceeding 6.5% (OR = 3.87, p < 0.001). The prevalence of abnormal vitamin B12 levels in individuals with DN was 63% (p = 0.19), indicating that the finding was not statistically significant. Within the group of individuals with DN being studied, 22% exhibited mild neuropathy, 34% displayed moderate neuropathy, and 44% had severe neuropathy. The DN individuals had significantly reduced ankle dorsiflexion in cases of severe NDS scores compared to those with mild to moderate NDS scores (p = 0.009). During the binary logistic regression analysis, it was shown that the duration of DM (OR = 1.73; p = 0.038) and FBG levels (OR = 2.87; p = 0.018) were determined as predictors for DN.

Conclusion: The findings of this study reveal that the duration of diabetes, age, literacy level, income, HbA1c, and FBG were substantially related to a higher likelihood of DN among diabetic patients.

## Introduction

Diabetic peripheral neuropathy (DN) is the most prevalent consequence of diabetes mellitus (DM) and a significant contributor to limb amputations and vulnerability to foot or ankle fractures. Atypical or diminished sensation in the toes that spreads to the foot and leg is the primary sign of DN [1]. The International Diabetes Federation estimated in 2017 that 425 million people worldwide suffer from diabetes. It is anticipated that the population will rise to 629 million by the year 2045. With 72,946,400 people overall in 2017, India ranked second among all countries in terms of the total number of people affected by DM [2, 3]. DM is identified as the fourth leading factor in worldwide disabilities [4]. The Indian Council of Medical Research-India Diabetes (ICMR-INDIAB) population-centered study indicated that the aggregate prevalence of diabetes in 15 Indian states was 7.3% [5]. DN can manifest in two forms: asymptomatic or symptomatic. When experiencing symptoms, it commonly manifests as burning pain, tingling sensations, and heightened sensitivity, causing discomfort. Approximately 50% of individuals will be asymptomatic and are less inclined to receive medical attention. In addition, primary care providers may neglect the necessity of conducting a foot evaluation for their patients.

With the growing incidence of DM, there is also an expected rise in its associated complications. The presence of DM and its associated consequences are anticipated to lead to higher rates of illness, death, and healthcare costs due to the heightened need for specialized medical attention [6]. DM is significantly linked to both micro- and macrovascular problems. Microvascular alterations result in the development of nephropathy, retinopathy, and neuropathy. DN is the predominant condition linked with diabetes, affecting approximately 50% of DM patients [7]. While DN usually presents as sensory and autonomic disorders, an expanding amount of research indicates that motor disorders in the ankle and knee can also be a significant symptom. Motor impairment manifests as muscle weakness, a decrease in muscle volume, and constraints on joint flexibility and range of motion (ROM), which eventually affect the individual's gait and movement of the entire body [8].

A sufficient dorsiflexion ROM in the ankle is essential for the proper execution of multiple functional processes, including walking, jogging, and climbing stairs [9]. These circumstances can have a detrimental effect on the overall well-being of the individuals involved [10]. The widespread presence of diabetic DN and its related variables among diagnosed DM patients in Kerala lacks sufficient evidence. The objective of this study was to evaluate the frequency of DN and its related risk variables among those with DM who were receiving medical care at a tertiary health institution in Kerala.

## Materials and methods

Study type

The study adopts a cross-sectional design.

Population

Conducted at a tertiary health center in Kerala, the study includes individuals diagnosed with type 2 DM. The diagnosis of diabetes was established by assessing the glycated hemoglobin A1c (HbA1c) level, which was found to be equal to or greater than 6.5%, and the fasting blood glucose (FBG) level, which was equal to or greater than 140 mg/dL. The subjects were assessed for neuropathy using a neuropathy disability score (NDS) [1]. Participants meeting inclusion criteria are aged 18 years or older and recently diagnosed with type 2 DM without a prior diagnosis or medication. Exclusion criteria include chronic lower back pain, previous neurological disorders, gestational diabetes, clinical signs of myelopathies, and the inability to partake in neurological examinations. Consequently, those who met the criteria for DM were classified into two groups: those with neuropathy and those without neuropathy. Individuals who smoked no less than 10 cigarettes per day for five years were categorized as smokers [11]. Non-smokers were defined as people who had abstained from smoking or had ceased smoking for at least two years before the commencement of the study.

Duration

Participants were progressively enrolled from December 2022 to November 2023.

Ethical approval

Approval was granted by the Institutional Ethics Committee under ethical number 009/12/2022/IEC/SMCH. Before enrollment, participants provided written informed consent.

Inclusion criteria required participants to be 18 years or older and recently diagnosed with type 2 DM without previous medication. The age group of 18 years or older was chosen because type 2 diabetes typically develops in adulthood, although there has been an increasing incidence of type 2 diabetes in younger age groups due to rising rates of obesity and sedentary lifestyles. Excluded were individuals with chronic lower back pain, previous neurological disorders, gestational diabetes, clinical indications of myelopathies, and those unable to participate in neurological examinations.

Data collection

Demographic data and medical history were obtained through a validated survey questionnaire. Neurological assessments employed an NDS, comprising four clinical tests on both feet.

A validated survey questionnaire was employed to gather demographic data and the medical history of DM and associated ailments. Participants were enrolled in the study consecutively until the target sample size was reached.

Subsequently, a meticulous inspection of the feet was conducted in a well-illuminated room. A dermatological, musculoskeletal, neurological, and vascular assessment was conducted. The feet were examined for the presence of alopecia, malformed nails, xerosis, fissures, ulcers, regions of erythema, and previous cicatrices as part of the dermatological evaluation. The musculoskeletal assessment documented the existence of muscle atrophy and abnormalities, including claw toes, the Charcot joint, and the large metatarsal head.

The neurological assessment was conducted using the NDS, which included four clinical tests performed on both feet. Before the assessment, the process was elucidated, and the tests were administered on the patient's hand. Throughout the examination, the patient kept their eyes closed. The assessment of each test was conducted using a point-based system to determine the overall disability score. The clinical tests were conducted as follows:

Pressure sensation was evaluated by applying a 10g monofilament to multiple locations on the sole, including the plantar base of the big toe, the 2nd and 5th toes, and the heel, while avoiding areas with calluses. Additionally, other bony prominences such as the metatarsal heads may have been tested for pressure sensation. Vibration perception was assessed by applying a 128-Hz tuning fork to various bony prominences such as the hallux of the big toe. Pain perception was evaluated by applying a pinprick to different areas, including the proximal area of the big toe, with sufficient pressure to slightly depress the skin.

In a foot with hypoesthesia, the monofilament test to assess pressure sensation was used. Pain perception was evaluated in the proximal area of the big toe with sufficient pressure to slightly depress the skin. The findings were categorized as "present" when the individual could discern the intensity of the pinprick and "absent" when the patient failed to perceive it. The three sensations were assigned a score of 0 if they were present and typical and a score of 1 if they were absent, decreased, or unknown. The Achilles deep tendon reflex was assessed utilizing a conventional patellar hammer technique. It was evaluated as follows: 0 if present, indicating normal function; 1+ if present with reinforcement; or 2+ if absent. The score for the investigated foot would be doubled if there had been an earlier amputated foot. The NDS has a score ranging from 0 to 10, which can also be utilized for evaluating the degree of severity of DN. The neuropathy disability was categorized into three severity levels: mild if the scores range between three and five, moderate when the scores are between six and eight, and severe when the scores are between nine and ten [12]. All research participants underwent laboratory examinations. The tests conducted encompassed a blood lipid profile, HbA1c, and vitamin B12 complex concentrations.

Sample size

The sample size was calculated using the formula: N = Z^2^p(1 - p)/E^2^, where Z = 1.96 (standard normal deviance at a 95% confidence interval) and E = 0.05 (desired precision). The study included 200 individuals with diagnosed DM, 100 in each group.

Data analysis

IBM SPSS Statistics for Windows, Version 25 (Released 2017; IBM Corp., Armonk, New York) facilitated data input and analysis. Descriptive statistical analysis presented underlying factors, with quantitative parameters shown as median and range, and qualitative variables as frequencies and percentages. Risk factors were analyzed via the chi-square test, with odds ratios (OR) and corresponding 95% confidence intervals (CI) calculated. A p-value < 0.05 indicated statistical significance. Binary logistic regression, employing the Backward-Wald method, identified predictors for DN occurrence.

## Results

A total of 256 DM patients were screened, and 56 subjects were excluded as they were not willing to give consent and exhibited other complications of DM. Thus, the study encompassed 200 DM patients, consisting of 120 males and 80 females. The longer a patient had DM, the higher the prevalence of DN, and this variance in percentage was of statistical significance (p<0.009) with an OR of 9.246. DN was not shown to be linked with gender, smoking history, alcohol use, hypertension, or familial history of DM (p > 0.05). A total of 114 participants (57%) reported having a positive family history of DM. Out of the total subjects, 159 (79.5%) were married and 34 (17%) were single. The analysis of the frequency distribution revealed that the results were not statistically significant. A statistically significant 40.5% of participants graduated, compared to 119 (59.5%) with only higher secondary education or less (OR = 2.042; p = 0.014). Approximately 107 (53.5%) of individuals earned an income under two lakhs, and this disparity was deemed statistically significant (p = 0.003) with an OR of 2.357 (Table 1).

**Table 1:** Sociodemographic traits and clinical history of the study sample p-value <0.05 was considered significant. N: number of subjects, DM: diabetes mellitus.

Sociodemographic traits		DM with neuropathy, N = 100	DM without neuropathy, N = 100	Total, N = 200 (%)	p-value
Age (years)	41-50	20	48	68 (34)	0.001
	51-60	42	31	73 (36.5)	
	61-70	38	21	59 (29.5)	
Gender	Males	55	65	120 (60)	0.149
	Females	45	35	80 (40)	
Duration of DM	≤5 years	15	62	77 (38.5)	0.001
	>5 years	85	38	123 (61.5)	
Marital status	Single	16	18	34 (17)	0.853
	Married	81	78	159 (79.5)	
	Separated	3	4	7 (3.5)	
Literacy level	Graduated	32	49	81 (40.5)	0.014
	Higher secondary or lower	68	51	119 (59.5)	
Income	>2 lakhs	36	57	93 (46.5)	0.003
	≤2 lakhs	64	43	107 (53.5)	
Familial history of DM	Yes	62	52	114 (57)	0.153
	No	38	48	86 (43)	
History of smoking	Present	60	58	118 (59)	0.774
Alcohol consumption	Present	58	50	108 (54)	0.256
Hypertension (mmHg)	Present	64	53	117 (58.5)	0.114

A large percentage of subjects were prescribed metformin, either as a standalone treatment or in combination with an additional oral hypoglycemic agent. In the group of individuals being studied, 53 (26.5%) of them experienced a decrease in pressure sensation, 47 (23.5%) had a decrease or absence of vibration perception, 48 (24%) had an absence or decreased pinprick response, and 46 (23%) had an absent ankle reaction. Table 2 provides a comprehensive overview of the assessment procedures for dermatological, musculoskeletal, neurological, and vascular conditions.

**Table 2:** A comparison of clinical characteristics of the study participants p-value <0.05 was considered significant. N: number of subjects, DM: diabetes mellitus.

Symptoms	DM with neuropathy, N = 100	DM without neuropathy, N = 100	Total, N = 200 (%)	λ^2^	p-value
Any 1 or more symptoms	92	13	105 (52.5)	125.13	<0.0001
Burning	28	9	37 (18.5)	11.97	0.001
Pricking	22	7	29 (14.5)	9.07	0.003
Pain	21	6	27 (13.5)	9.63	0.002
Numbness	31	11	42 (21)	12.06	0.0005
Tingling	29	15	44 (22)	5.71	0.02
Dermatological and musculoskeletal assessment					
Absent hair	21	10	31 (15.5)	4.62	0.03
Deformed nails	19	7	26 (13)	6.37	0.011
Fissured skin	27	9	36 (18)	10.98	0.0009
Old healed scar	14	1	15 (7.5)	10.38	0.001
Neurological assessment					
Monofilament test – pressure sensation absent or reduced	35	18	53 (26.5)	7.42	0.006
Vibration test – vibration perception absent or reduced	38	9	47 (23.5)	23.39	<0,0001
Pain perception – pinprick test	36	12	48 (24)	15.79	<0.0001
Achilles deep tendon reflex	31	15	46 (23)	7.23	0.007
Vascular assessment					
Dorsalis pedis artery – pulsations absent	6	4	10 (5)	0.11	0.75
Posterior tibial artery – pulsations absent	5	4	9 (4.5)	0	1

Out of the individuals involved in the study, 105 (52.5%) were found to exhibit one or more symptoms that indicated the presence of DN. Out of the individuals who had been diagnosed with DN, 92% experienced symptoms. The study found that the most commonly reported symptoms were tingling (n = 44; 22%), numbness (n = 42; 21%), burning (n = 37; 18.5%), pricking (n = 29; 14.5%), and pain (n = 27; 13.5%).

The incidence of DN among the study subjects based on the pertinent laboratory parameters is presented in Table 3.

**Table 3:** A comparison of laboratory investigations of the study sample BMI: body mass index, TC: total cholesterol, TGL: triglycerides, HDL: high-density lipoprotein, LDL: low-density lipoprotein, FBG: fasting blood glucose, HbA1c: glycated hemoglobin A1c, N: number of subjects, DM: diabetes mellitus. p-value <0.05 was considered significant.

		DM with neuropathy, N = 100	DM without neuropathy, N = 100	Total, N = 200 (%)	p-value
BMI (kg/m^2^)	18-24.99	36	45	81 (40.5)	0.195
	≥25	64	55	119 (59.5)	
TC (mg/dL)	≤200	41	46	87 (43.5)	0.476
	>200	59	54	113 (56.5)	
TGL (mg/dL)	≤150	44	44	88 (44)	1
	>150	56	56	112 (56)	
HDL (mg/dL)	≤35	60	70	130 (62)	0.138
	>35	40	30	70 (35)	
LDL (mg/dL)	≤135	40	39	79 (39.5)	0.885
	>135	60	61	121 (60.5)	
FBG (mg/dL)	≤140	20	53	73 (36.5)	0.001
	>140	80	47	127 (63.5)	
HbA1c (%)	≤6.5	24	55	79 (39.5)	0.001
	>6.5	76	45	121 (60.5)	
B_12_ (pg/mL)	>200	37	40	77 (38.5)	0.663
	≤200	63	60	123 (61.5)	

The study subjects had a median BMI of 28.88 kg/m^2^, ranging from 23.47 to 39.04 kg/m^2^. There was no statistically significant variation in BMI across the groups. A strong association was found between DN and glycemic markers, namely, FBG levels exceeding 140 mg/dL (OR = 4.511, p < 0.001) and HbA1c levels exceeding 6.5% (OR = 3.87, p < 0.001). The bivariate analysis of the laboratory findings revealed that 121 (60.5%) of those diagnosed with neuropathy had high HbA1c levels, with a statistically significant difference (p < 0.001). The prevalence of abnormal vitamin B12 levels in individuals with DN was 63% (p = 0.19), indicating that the finding was not statistically significant. A standard lipid profile was typically associated with a reduced risk of DN; however, the findings failed to reach statistical significance.

**Figure 1:**
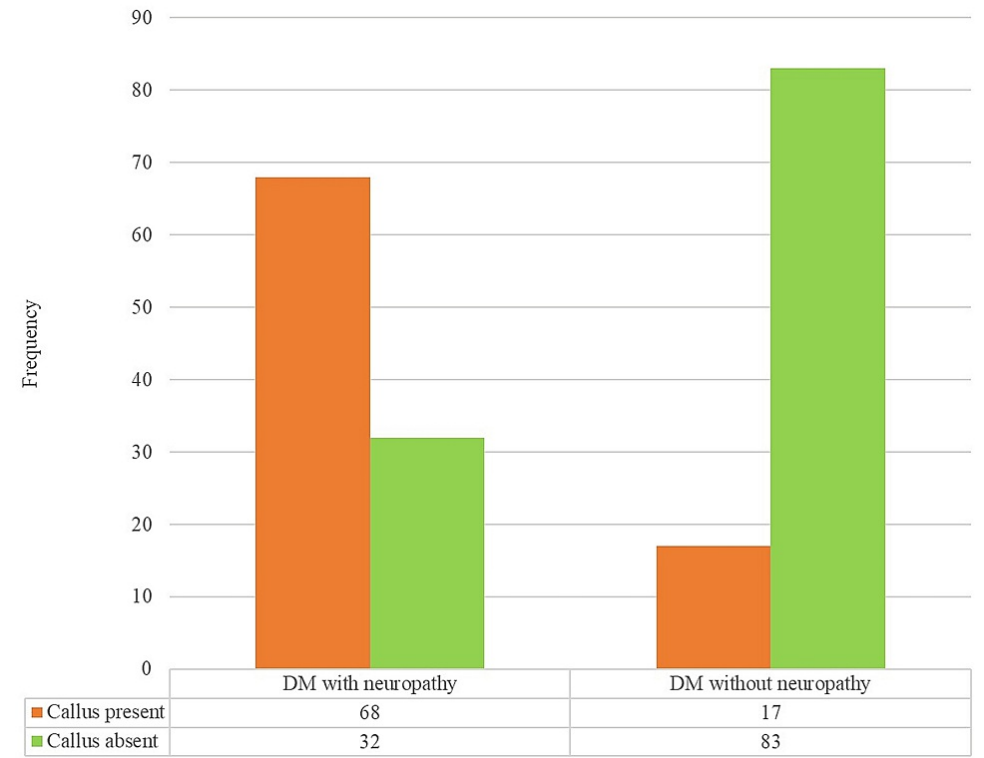
Frequency distribution of the presence of callus in the study sample

A notable discrepancy in the occurrence of calluses was observed between individuals with and without neuropathy (p < 0.001) (Figure 1).

Among DN patients, 68 individuals exhibited callus, whereas only 17 individuals with DM without neuropathy exhibited callus.

Table 4 demonstrates the correlation between the severity of neuropathy disability and the decrease in ankle dorsiflexion ROM.

**Table 4:** A comparison of the range of motion of ankle dorsiflexion and degree of severity of neuropathic disability p-value <0.05 was considered significant. N: number of subjects, SD: standard deviation.

Variable		Ankle dorsiflexion range of motion		
Neuropathic disability	N = 100	Mean ± SD	F-test	p-value
Mild	22	8.32 ± 4.93	4.93	0.009
Moderate	34	6.08 ± 3.08		
Severe	44	5.72 ± 2.2		

Within the group of individuals with DN being studied, 22% exhibited mild neuropathy, 34% displayed moderate neuropathy, and 44% had severe neuropathy. The current study found that diabetic individuals with neuropathy had significantly reduced ankle dorsiflexion in cases of severe NDS scores compared to those with mild to moderate NDS scores (p = 0.009).

A binary logistic regression analysis was conducted to examine the relationship between the prevalence of DN and variables including duration of DM, income, education status, HbA1c, and FBG. These findings indicate that a disease duration of more than 5 years (p < 0.001) and higher FBG levels (p = 0.033) are significantly associated with DN, as shown in Table 5.

**Table 5:** Logistic regression analysis of the associated factors of diabetic neuropathy among the study sample FBG: fasting blood glucose, HbA1c: glycated hemoglobin A1c, DM: diabetes mellitus, B: beta coefficient, SE: standard error, λ^2^: likelihood ratio chi-square, df: degrees of freedom, Exp (B): exponentiation of the beta coefficient, 95% CI: 95% confidence interval.

Variables	B	SE	λ^2^	df	p-value	Exp (B)	95% CI for EXP(B)	
Duration of DM (years)	-2.020	0.365	30.558	1	0.001	0.133	0.065	0.272
Income	-0.189	0.854	0.049	1	0.825	0.828	0.155	4.409
Literacy level	-0.268	0.857	0.098	1	0.754	0.765	0.143	4.100
FBG (mg/dL)	-1.470	0.691	4.524	1	0.033	0.230	0.059	0.891
HbA1c (%)	0.335	0.688	0.237	1	0.627	1.397	0.363	5.378
Constant	1.311	0.268	24.010	1	0.001	3.710		

## Discussion

The current investigation was conducted among individuals with DM who were receiving care at a specialized medical facility to ascertain the contributing factors to its occurrence. The study included a group of 200 participants diagnosed with DM. The diagnosis of DN involved the use of a 10-gram monofilament and VibraTip to measure the vibration perception threshold and assess the ankle reflex. During the binary logistic regression analysis, it was shown that the duration of DM (OR = 1.73; p = 0.038) and FBG levels (OR = 2.87; p = 0.018) were determined as predictors for DN. Based on the ICMR-INDIAB study, it appears that the diabetes problem is becoming more stable in the wealthier states of India. The number of people with prediabetes is decreasing, which suggests that the prevalence of DM may level off in the coming decades in these areas. Nevertheless, the transmission of the disease to socioeconomically deprived subgroups of the population is a significant cause for concern in India, as the majority of medical expenses for DM are paid directly by individuals [5].

The prevalence of the condition was considerably greater among males (46.2%) compared to females (44.4%), although this difference did not have statistical significance. These results align with the findings of Bansal et al. [13] and Jasmine et al. [3], while there are investigations that indicate males have a greater susceptibility to developing DN as opposed to females [14, 15]. Van der Meer et al. observed that individuals with DM who had a limited educational background experienced more severe consequences [16]. An investigation conducted in India found that individuals with lower levels of schooling who have DM have a higher occurrence of microvascular events than people with a higher level of education [17]. These findings corroborate our research and can be attributed to the notion that individuals with a higher level of education possess greater awareness regarding DM and its associated problems. They are more inclined to embrace and follow modifications in their lifestyles, medications, and suitable food practices [3].

Liu et al. conducted a meta-analysis to examine the risk indicators associated with DN. The analysis comprised documentation from China, India, Bangladesh, and Kuwait. This study found that HbA1c is a risk marker for DN, which is consistent with the current study [18]. According to a study conducted by Kasper et al., it has been found that extended periods of unregulated high blood sugar levels have been linked to DN as well as other problems affecting micro- and macrovasculature [19]. Ishibashi et al. did a study that showed people who have just been diagnosed with type 2 diabetes can delay the onset of microvascular problems like neuropathy and nephropathy by strictly controlling their blood sugar levels [20]. This indicates that the glycemic state has an impact on the development and advancement of problems associated with diabetes [3].

The current recommendations for addressing and preventing issues related to diabetic feet involve the regulation of diabetes, comprehensive foot care, patient education, and independent foot care management. Exploring alternative rehabilitation methods, such as physical activity, has shown positive effects on diabetic foot outcomes. Specifically, it has been found to enhance the rate of nerve conduction in the lower extremities, improve skin sensitivity, and increase intraepidermal neuronal density. These improvements can potentially slow down the progression of DN, prevent skin damage, and defer the occurrence of ulcers [21]. Neuromusculoskeletal changes frequently occur in DN patients. Implementing interventions such as muscle strengthening, stretching, balance exercises, and gait rehabilitation can be advantageous in averting the development of calluses, foot ulcers, and the need for amputation. These interventions also mitigate the risk of falls and enhance levels of daily exercise and overall quality of life. Ultimately, these measures contribute to a decrease in mortality and complication rates [22].

Diabetic individuals who have decreased glucose tolerance and do not show any obvious signs of neuropathy experience the development of intense neuropathic symptoms and impairment of tiny nerve fibers. This presents a difficulty in evaluating the condition and therefore necessitates an in-depth clinical assessment of all diabetes patients, regardless of the presence of neuropathy manifestations. Conducting screenings for DN may be an economically feasible way to reduce the occurrence of diabetic foot ulcers. Advanced age, extended duration of diabetes, and inadequate glycemic control are widely acknowledged as significant risk factors for DN. Additionally, cigarette smoking, retinopathy, obesity, high blood pressure, elevated cholesterol levels, and microalbuminuria have also been identified as possible contributory indicators [1]. The investigation undertaken by Bansal et al. revealed a noteworthy correlation with increasing age, duration of diabetes, and glycemic level [13]. The prolonged duration of diabetes and inadequate regulation of blood sugar levels lead to the buildup of glycosylation end products, reactive oxygen species, and impairment of the endothelial cells. These factors play a crucial role in the pathological process of DN [23]. It has been confirmed that the prevalence of DN increases dramatically with every decade of age. Research conducted in Nigeria found that the average age was 54.8 ± 12.1 [24]. In Bangladesh and India, the average age was 54.8 ± 12.1 [25] and 51.3 ± 12.3 [26], respectively. Nevertheless, the prevalence was greater in China (69.6±9.5) [27] and Sri Lanka (62.1 ± 10.8) [28] due to their research group consisting of individuals aged 50 years and beyond [1].

Restricted ankle joint ROM is a frequent issue among individuals with DN, likely due to a buildup of advanced glycation end products and the thickening of the Achilles tendon. Goniometric tests on people with deformities in the metatarsophalangeal joint have shown that they have a limited ROM in the dorsiflexion of the ankle joint [29]. The neurological symptoms linked to vitamin B12 deficiency may be mistakenly identified as symptoms of diabetic neuropathy in diabetic individuals who regularly use metformin over a long period [30]. Nevertheless, in the current investigation, we assessed the vitamin B12 levels in both groups and determined that they were not statistically significant.

There are certain constraints in the present investigation. Firstly, it is crucial to note that this study was conducted in a referral healthcare context, which means that the findings may not apply to other settings or populations. Furthermore, the nerve conductivity investigations, which are considered the most accurate method for diagnosing DN, were not administered due to logistical and financial limitations. This may have resulted in an underestimation of the prevalence of DN. DN can hamper the achievement of therapeutic goals in muscles affected by nerve damage, although there is a dearth of studies in this specific field. Further investigation is necessary to determine if a targeted intervention focused on the foot can impact the ROM in ankle dorsiflexion, the strength of intrinsic foot muscles, and the extension of the MTPJ during movements to enhance the alignment of the joint [29].

An advantageous aspect of this study is the utilization of simple, non-intrusive, cost-effective, and expedient techniques to assess DN. The simplicity of conducting these point-of-care examinations in a clinical environment makes them effective screening tools at the primary healthcare level. However, the process of screening for neuropathy is not extensively utilized in general care practices. Healthcare providers should promptly evaluate for DN when patients exhibit symptoms or have indicators of susceptibility to the condition. Regular foot examinations, such as visual inspection of the feet, assessment of sensation, examination of pulses, and testing of reflexes, should be conducted for diabetic patients visiting primary health centers. This will aid in the early detection and timely treatment of patients, thereby reducing the risk of ulcers and amputations.

The study's limitations include its confinement to a specialized medical facility, potentially limiting the generalization of findings. The absence of nerve conductivity investigations due to logistical and financial constraints may have led to underestimation of DN prevalence, while the lack of comprehensive diagnostic tests could have overlooked individuals with early-stage neuropathy. Furthermore, the study's scope did not thoroughly evaluate the impact of targeted interventions on foot functionality or consider the possibility of misidentifying neuropathy symptoms related to vitamin B12 insufficiency. Additionally, the study lacked a comprehensive longitudinal analysis of DN progression and its associated factors over time and did not address the feasibility of implementing foot examination practices in general care settings. These limitations highlight the need for future research to incorporate more diverse populations, comprehensive diagnostic measures, longitudinal assessments, and considerations of practical implementation challenges in healthcare settings.

## Conclusions

The findings of this study revealed that the duration of diabetes, age, literacy level, income, HbA1c, and FBG were substantially related to a higher likelihood of DN among diabetic patients. However, BMI, smoking, and lipid parameters did not show any indication of enhancing the risk of DN. Additionally, the duration of DM and FBG can be regarded as independent predictive factors that are linked to DN. Furthermore, the current study found that diabetic individuals with neuropathy had significantly reduced ankle dorsiflexion in cases of severe NDS scores compared to those with mild to moderate NDS scores. The findings offer an empirical foundation for enhancing insight into the etiology of DM accompanied by DN as well as the outcomes of preventative measures.
